# Monthly Summary

**Published:** 1889-11

**Authors:** 


### Monthly Summary.

---

A SUGGESTIVE CASE.—Mrs. C., aged about fifty, has been wearing a full set of teeth on rubber plate about twenty years. They were worn at this time till within the last two and a half years; during this time there has been considerable soreness at the posterior border of the upper plate; this was relieved by cutting away the border of the plate about two lines and rounding it off at the upper edge. There was also a little irritation of the mucous membrane at the buccal border of the same plate which was also relieved by dressing. The irritation at both these points was undoubtedly due to the settling, though very slow, of the alveolar ridge.

A greater and apparently more serious difficulty was found on the tongue on the left side and about opposite the first and second molars on the lower denture. This consisted of an ulcer of about a third of an inch in diameter, and a line in depth. Round about this there was considerable induration. It had been in this condition for more than two years, and all this time undergoing treatment by a physician, or physicians rather, for several had treated it. The statement was made that it was probably malignant and would necessitate the removal of a part, if not all of the tongue. The patient had been for a considerable time in great trouble about it and was much depressed.

She was questioned, as to whether the tongue was ever caught between the teeth, to which she replied that she was not aware that there was anything of the kind. Upon examination it was found that the molars of both the upper and lower dentures of the left side had been ground quite smooth on the masticating surfaces and very squarely, so that the inner edges came very accurately together, quite to the lingual surfaces of these teeth, and it was found that the tongue was in masticating, constantly, though imperceptibly, caught in this close-fitting bite; thus inducing and continuing the ulcer on the tongue. The angles of these were now rounded off and dressed so that the tongue was not caught. Immediate improvement of the affected part resulted and in a short time was entirely well.

Two points are brought to notice by this case: the first is that care should be taken by dentists in the construction of artificial dentures, whether full or partial sets, to avoid all points of irritation to the soft parts either by roughness of any part, or by unequal pressure, especially at the edges of the plates, or as in the case above described, by biting and bruising the soft parts in the occlusion of the teeth; and secondly more care should be exercised by both dentists and physicians, and especially the latter, in respect to mouth affections, and especially those connected with the teeth, as to diagnosis and prognosis. The case above given was treated by a physician for about two years under the impression that it was rapidly tending to malignancy, and he was of the opinion that it had already attained that condition, and that nothing short of the removal of a part, if not the whole of the tongue would prevent a fatal result. In this case two or three physicians had the same opinion. Two dentists examined it, and were quite befogged as to the cause or nature of the affection. Had there been in this case a favorable condition for the development of cancer or malignant tumors, such disease might have taken place, but that it had not yet assumed that form was manifest by the results; and still further, the more obvious indications of malignancy were not present.

It is well that due caution be always exercised in the examination and pronouncing on affections arising from diseased teeth, for they are often very obscure, treacherous and persistent. Error has been too often committed in prematurely announcing malignancy, but on the other hand of denying its presence when strong evidence was present to that effect. Malignant diseases should be studied and better understood by all who in a medical or surgical aspect may have any thing to do with them.—*Dental Register.*

---

AN IMPROVED WAY OF USING THE HYPODERMIC METHOD.—Dr. P. G. Udell, of Spencerport, N. Y., writes: "Every physician who employs the hypodermic method is aware of the fact that painful swelling and abscess not infrequently follow its use. This undoubtedly arises from the introduction of septic matter—because the method of antisepsis has not been rigidly applied to this simple surgical proceeding. The sources of infection are numerous. The syringe may be unclean; the needle may be foul; the hypodermic tablet may be composed of materials that undergo fermentation; the water used as a solvent may be impure; the spoon employed to prepare the solution in may furnish the septic matter, etc. I have for some time used a method whereby the above dangers may be positively avoided. In one of the compartments of my syringe-case I carry two small test tubes, nested; the inner one is converted into a vial by closing the open end with a cork, and contains my hypodermic needles immersed in absolute alcohol. This agent sterilizes any septic matter that may be on the needles, and does away with the necessity of using a wire for maintaining the patency of the same; there is no rusting, and the needle is always aseptic. I frequently cleanse the syringe with a five per cent. carbolic acid solution. Having occasion to use it, I free the needle of alcohol by passing through it water that has been boiled. I now dissolve one of Wyeth's tablets in a few minims of boiled water, and for this purpose carry the second test-tube, in which water may be boiled quickly over any flame that may be convenient, a match answering the purpose very well. After the solution is sufficiently co led, is is drawn into the springe, and is really for use. The above plan may seem somewhat fussy in detail, but in practice is exceedingly simple, and, if followed as directed, should relieve the physician of those mishaps so annoying to himself and patient. If some instrument maker will materialize this idea, it will involve but slight increase in size of the pocket-case now in use."—*Medical Record.*

---

THE MORTALITY AMONG NURSES.—The question as to the liability of nurses to infection while in attendance on phthisical patients is an important one and has not hitherto been studied with the attention it merits. The result of a careful inquiry into the increase in Germany, where the nurses are largely drawn from religious corporations, shows that while the mortality from tuberculosis among the sisters who remain in the convent is from one-seventh to one-sixth of the total mortality, among the nursing sisters the proportion amounted to nearly two-thirds. The death-rate from tuberculosis among the nursing sisters attains its maximum between the thirtieth and fortieth year, and then steadily declines. Compared to the deaths from this cause among the population of the towns in which they live, the mortality from all causes among nurses between the ages of fifteen and twenty is four times as great as outside, between twenty and thirty three times as great, and from thirty to forty twice as great, the explanation being the prevalence of tuberculosis among nurses, the death-rate from this source alone being nine times as great as among the general population. Infectious diseases levy an excessive tribute on nurses up to the age of forty, after which time deaths from this cause become infrequent. It appears, moreover, that nurses enjoy comparative immunity from infection during the first six months' service, but the proportion of disease and death then rises steadily for three years. Of course the figures only represent an average, and do not apply to particular hospitals and institutions, the hygienic conditions of which necessarily vary very considerably. A return drawn up upon reliable data would be of very great advantage from several points of view, and perhaps it may, at some future time, be forthcoming.—*Medical Press and Circular.*

THE COCOANUT AS A TÆNIACIDE.—Professor Parisi, of the University of Athens, accidently made the discovery recently that the cocoanut possesses anthelmintic properties. He ate the fruit of the nut after having drank the milk. Two hours later he began to feel somewhat nauseated and had a slight diarrhœa. The following morning he passed at stool a dead tape-worm with its head. He was then in Abyssinia and inquired of the natives whether they made use of the cocoanut for this purpose, but none of them had ever known it to be so used. Upon Dr. Parisi's return to Athens he used the remedy in a number of cases and found it very efficacious. It is preferable to other tæniacides, he says, in that no preparatory treatment is necessary. In all cases the entire worm, including its head, was passed at stool on the morning following the administration of the cocoanut.—*Schmidt's Jahrbucher.*

---

BEER COMPARED WITH OTHER ALCOHOLICS.—For some years a decided inclination has been apparent all over the country to give up the use of whiskey and other strong alcoholics, using as a substitute beer and other compounds. This is evidently founded on the idea that beer is not harmful, and contains a large amount of nutriment; also that bitters may have some medical quality which will neutralize the alcohol which it conceals, etc. These theories are without confirmation in the observation of physicians. The use of beer is found to produce a species of degeneration of all the organs. Profound and deceptive fatty deposits, diminished circulation, conditions of congestion, and perversion of functional activities, local inflammations of both liver and kidneys are constantly present. Intellectually a stupor amounting to almost paralysis arrests the reason, changing all the higher faculties into a mere animalism, sensual, selfish, sluggish, varied only with paroxysms of anger that are senseless and brutal. In appearance, the beer-drinker may be the picture of health, but in reality he is most incapable of resisting disease. A slight injury, a severe cold, or a shock to the body or mind, will commonly prove acute disease, ending fatally. Compared with inebriates who use different kinds of alcohol, he is more incurable, and more generally diseased. The constant use of beer every day gives the system no recuperation, but steadily lowers the vital forces.

Recourse to beer as a substitute for other forms of alcohol merely increases the danger and fatality.—"Scientific American."

